# Parallel genetic and phenotypic differentiation of *Erigeron annuus* invasion in China

**DOI:** 10.3389/fpls.2022.994367

**Published:** 2023-01-04

**Authors:** Yuan-Yuan Liu, Qin-Fen Yang, Zhen Li, Zhi-Xiang Zhou, Xue-Ping Shi, Yong-Jian Wang

**Affiliations:** College of Horticulture and Forestry Sciences/Hubei Engineering Technology Research Centre for Forestry Management Key Laboratory, Huazhong Agricultural University, Wuhan, Hubei, China

**Keywords:** geographical population, growth performance, coefficient of variation, genetic diversity and variation, dominance ecotype

## Abstract

**Introduction:**

The factors that determine the growth and spread advantages of an alien plant during the invasion process remain open to debate. The genetic diversity and differentiation of an invasive plant population might be closely related to its growth adaptation and spread in the introduced range. However, little is known about whether phenotypic and genetic variation in invasive plant populations covary during the invasion process along invaded geographic distances.

**Methods:**

In a wild experiment, we examined the genetic variation in populations of the aggressively invasive species *Erigeron annuus* at different geographical distances from the first recorded point of introduction (FRPI) in China. We also measured growth traits in the wild and common garden experiments, and the coefficient of variation (CV) of populations in the common garden experiments.

**Results and discussion:**

We found that *E. annuus* populations had better growth performance (i.e., height and biomass) and genetic diversity, and less trait variation, in the long-term introduced region (east) than in the short-term introduced region (west). Furthermore, population growth performance was significantly positively or negatively correlated with genetic diversity or genetic variation. Our results indicate that there was parallel genetic and phenotypic differentiation along the invaded geographic distance in response to adaptation and spread, and populations that entered introduced regions earlier had consistently high genetic diversity and high growth dominance. Growth and reproduction traits can be used as reliable predictors of the adaptation and genetic variation of invasive plants.

## Introduction

Alien plant invasions are considered to be a major threat to native biodiversity and ecosystems worldwide (Richardson and Pyšek, 2006; Richardson et al., 2007; van Kleunen et al., 2015; Pyšek et al., 2020; Diagne et al., 2021). The factors that determine the growth and spread advantages of an alien plant during the long-term invasion process remain open to debate (Bossdorf et al., 2005; Beerli and Palczewski, 2010; Wang et al., 2017; Xu et al., 2019; Sun et al., 2020). Once alien plant species emerge in a new range, their genetic diversity and differentiation are likely to be closely related to population growth adaptation during the invasion process (Colautti et al., 2010; Qiao et al., 2019; Zhang et al., 2021). Moreover, as predicted by the evolution of the increased competitive ability hypothesis, alien plants often have the potential for evolutionary adaptations to new habitats, which may allow them to spread and invade successfully (Bossdorf et al., 2005; Sax et al., 2007; Prentis et al., 2008; LaForgia et al., 2020; Wang et al., 2022). Plant invaders might often undergo multiple evolutionary adaptations to different environmental stresses and selection pressures in the introduced range (Lee, 2002; Richards et al., 2006; Colautti et al., 2010; Hendry, 2016), as was the case with the aggressive invaders *Mimulus guttatus* (in Europe) and *Tamarix ramosissima* and *Helianthus tuberosus* (North America) (Sexton et al., 2002; Bock et al., 2018; Querns et al., 2022). Evolutionary adaptations promote the spread of invasive plants due to increasing competitiveness (such as accumulating more biomass, growing taller, or producing more flowers and seeds) through genetic variation (Lee, 2002; Lambrinos, 2004; Sax et al., 2007; Prentis et al., 2008). However, few studies have tested the relationship between population-level genetic and phenotypic variation of invasive plants and rapid adaptation in long-term introduction processes.

Regional expansion of invasive plant populations along geographical distance gradients provides opportunities for genetic variance and the potential for evolutionary adaptation of populations to strong selection (Lee, 2002; Xu et al., 2010). The processes leading to population-level genetic variation directly affect survival, reproduction, and other fitness-related phenotypes of invading plants (Garcría-Ramos and Rodríguez, 2002; Ohadi et al., 2016). Once invasive plants spread in large geographic areas, genetic variation (such as allelic diversity) within populations might be reduced and result in high genetic differentiation based on the dominant population effect (Walker et al., 2009). Indeed, the alternative genetic variability of invading populations is commonly believed to be driven by both natural selection and genetic drift, which might modify their tolerance or behavior (Hall and Willis, 2006; Sambatti and Rice, 2006; Stroup, 2015). On the other hand, recent studies have shown that environmental variation along geographic gradients can also lead to the growth adaptation differentiation of invasive plants (Colautti and Barrett 2010, Colautti and Barrett, 2013). Geographic gradients or geographic distance in different regions not only reflected growth and resource competition between alien and native plants but also exhibited the invasion process (i.e., invading periods of earlier invasion or more recent invasion) of alien plants and their phenotypic adaptation and differentiation (Griffith and Watson, 2006; Eckert et al., 2008). Thus, whether an alien plant species can spread and invade on a larger scale is largely dependent on growth advantage and phenotypic adaptation, and finally, through environmental selection and self-evolution, a dominating genotype with low genetic variation is formed (Alpert et al., 2000; Li et al., 2014). Evolutionary adaptation in invasive species arose in response to shifts in environmental conditions and genetic variation. However, the effects of environmental conditions (i.e., different geographic populations) in different regions and genetic variation, and their correlation with phenotypic variation in invasive plant species, have rarely been discussed.

Previous studies showed that the genetic diversity of invasive plants was very different between populations with different geographic distances (Wang et al., 2012; Havrdová et al., 2015; Zhang et al., 2016; Qiao et al., 2019), and might lead to significantly adaptive traits along geographic distances (i.e., as a result of invading time, Griffith and Watson, 2006; Eckert et al., 2008). Invasive plants commonly suffered long-term adaptation and multiple or single evolutions at the point of introduction (FRPI, i.e., the first collected location). Thus, in the earlier invasion ranges, i.e., FRPI, the invader was likely to have high genetic diversity and high growth dominance (i.e., grew taller and produced more biomass) than the later or recent invasion ranges (Sultan et al., 2013; Jeschke and Heger, 2018; Egbon et al., 2020; Querns et al., 2022). Genotypes of invasive plants have dramatic effects on performance during introduction and colonization (Taylor and Keller, 2007; Keller et al., 2012). Therefore, the variation in genetic diversity and differentiation of invasive plants with invasion time or geographic distance can be used to understand the adaptation and invasion mechanism of alien plant species.

Invasive plants respond to evolving adaptations not only through phenotypic changes in parents but also in offspring. The transgenerational effects allow organisms to conserve long-term environmental adaptation between generations and enhance offspring performance (Dong et al., 2019). By removing complex field environmental effects and underlining population evolutionary adaptation in offspring, common garden experiments can characterize the genetic variation in plant phenotypic performance, e.g., plant height (Andalo et al., 2005; Wei et al., 2019; Depardieu et al., 2020). Therefore, a combination of field and common garden experiments can provide novel insights into how plant phenotypes react to genetic influences over time.

*Erigeron annuus* L. (Asteraceae) is an annual or biennial plant. It is an apomictic plant, producing large numbers of minute seeds that are genetically identical to the mother plant. Thus, it can maintain the dominant performance of the mother plant for a long time, and its genetic diversity is reduced within populations; however, most populations contain several dominant genotypes, suggesting that sexual reproduction does occur occasionally (Edwards et al., 2006; Trtikova et al., 2011; Ma and Li, 2018). Owing to its strong apomictic reproductive ability and rapid dispersal, it can invade local ecosystems across broad anthropogenic habitats in China, particularly grasslands and farmlands (Wang et al., 2010; Liu et al., 2022). Several studies have investigated the ecological adaptability, interspecific competition, reproductive and biological characteristics, and phenotypic plasticity of *E. annuus*, as well as its genetic diversity and genotypic differentiation (Edwards et al., 2006; Trtikova et al., 2010; Trtikova et al., 2011). However, whether phenotypic and genetic variation in *E. annuus* populations covary in the invasion process in China along the geographic distance of the invasion remains unclear.

In a wild experiment, we examined the genetic variation of *E. annuus* populations at different geographical distances to the first recorded point of introduction (FRPI, i.e., the first collected invasive specimen of *E. annuus*) in China. We also measured the growth traits in the wild and in a common garden experiment, and analyzed the coefficient of variation (CV) of growth traits in nine geographical populations. Furthermore, we examined both the genetic and phenotypic differentiation and their relationships with the distance to the FRPI. We specifically addressed the following questions: (1) Do growth traits and their CV differ among populations at different geographical distances in wild or common garden conditions, and respond to the geographical distances from the FRPI? (2) Do genetic diversity and variation decrease and differentiation increase in populations with distance from the FRPI? (3) Do phenotypic and genetic variation of populations covary in response to adaptation and spread, and can phenotypic traits be used as significant predictors of adaptation and genetic variation in invasive plants?

## Materials and methods

### Plant species

Individuals of *E. annuus* are invasive and widely distributed in anthropogenically disturbed habitats, including roadsides, grasslands, and farmlands (Wang et al., 2010). The first collected invasive specimen of *E. annuus* in China was from Shanghai in 1886, and the species now covers a wide range, with longitudes ranging from 95°E to 123°E (Wang et al., 2010; Ma and Li, 2018).

In our study, we chose the populations according to geographic distance to the first recorded point of introduction (Shanghai in 1886, i.e., the zero point of geographic distance, FRPI), as we were interested in testing for variations between different geographic distances and invasion times. Therefore, the nine selected geographic populations had different distances (from 30 km to 1425 km) from Shanghai (FRPI), which were located in different cities: Jiaxing (JX, 30 km), Hangzhou (HZ, 133 km) and Wenzhou (WZ, 340 km) in the eastern region, Xianning (XN, 660 km), Wuhan (WH, 685 km), and Xiangyang (XY, 874 km) in the central region, and Chengkou (CK, 1230 km), Nanchuan (NC, 1373 km), and the main districts of Chongqing (CQ, 1425 km) in the western region (**Supplementary Table 1**; **Supplementary Figure 1**). In total, we chose three geographic populations (i.e., sites) in each region and selected four populations within each geographic population or site. For each population, we collected plants and seeds from four locations >500 m apart to increase the likelihood of sampling plants from different genotypes. The habitats of the populations were chosen on habitat-similar roadside farmlands. In total, we had 36 populations.

### Field survey and seeds collection

From July to August 2012, within each of four selected populations in each geographic population, five typical mother plants (the distance between two sample plants was at least 10 m) were selected, and the heights were measured during the flowering period. Then, we collected two to three fully developed and undamaged leaves from each plant in 50 ml tubes placed on ice for DNA extraction. The sampled leaves were air-dried and used for leaf biomass calculation of each sampled mother plant. Then, whole mother plants were harvested and brought to the laboratory, separated into envelopes, on the basis of whether they were root, stem, leaf, branch, and flower, and dried at 80°C for 72 h in an oven. The total biomass of the plants was the sum of the biomasses of the five parts. To compare the complex genetic difference and maternal effect in offspring between different *E. annuus* populations, the CV in traits was included in the analysis. The CV was calculated using the following formula:


CV = the standard deviation/the mean value for each morphological trait.

We also selected five other typical mother plants and sampled fresh leaves for DNA extraction. Then, we collected seeds from the top inflorescence of the plants at the mature seed stage. Each mature seed sample was taken directly from each mother plant and then brought back to the laboratory for preservation. All seeds were cleaned, air-dried, and stored at 4°C. These seeds were used in the common garden experiment. There were 360 samples in total (ten [i.e., five from mother plants related to growth traits and five from other mother plants related to seed collection and for common garden experiment] × four selected populations × nine geographic populations) for DNA extraction and microsatellite analyses.

### Sampling and DNA extraction

For each collected leaf sample, total genomic DNA was extracted from 50-100 mg of leaf material using either the CTAB method (Doyle and Doyle, 1987) or a DNeasy Plant Mini Kit (Qiagen, Valencia, CA, USA). The leaves were ground with a glass bead in 2 ml tubes for 3 min at an amplitude of 80 using a vibration mill (Retsch MM 2000). The ground material mixed with 300 μl of CTAB buffer with 5% 2-mercaptoethanol was incubated at 65°C for 30 min, extracted twice with 300 μl chloroform-isoamyl alcohol (24:1), precipitated with 100 μl of isopropanol, and washed with 250 μl of 70% ethanol. Finally, DNA was suspended in 50 μl of double-distilled water. DNA quality was assessed by electrophoresis on a 1% agarose gel. All extracted samples were stored at -20°C.

### Microsatellite analysis

Expressed sequence tag-microsatellite sequence (EST-SSR) markers developed for *E. annuus* were screened for transferability and polymorphism in *E. annuus*. Three of these were found to be sufficiently polymorphic **(Supplementary Table 2**). The polymorphic loci have been cross-amplified in related species (*Dendranthema morifolium* (Asteraceae) and *Lactuca sativa* (Asteraceae)). The markers were able to amplify corresponding DNA in the other two related species. Similar high cross-amplification was also observed in EST-SSR markers developed from *Solidago virgaurea* (Asteraceae), which were successfully transferred to the invasive species *Solidago canadensis* (Asteraceae) and *Solidago hispida* (Asteraceae) (Sakaguchi and Ito, 2014). PCRs were carried out in a total volume of 20 μl, consisting of approximately 50 ng/μl template DNA (4.0 μl), 10 × PCR buffer (2.0 μl), 2 mM dNTP mixture (2.0 μl), 100 pM primer pair (0.2 μl of each), and 2.5 U/μl Blend Taq (0.5 μl) (TOYOBO, Osaka, Japan). The PCR amplification conditions included initial denaturation at 94°C for 5 min; 40 cycles of 95°C for 50 s, 55°C for 30 s, and 72°C for 60 s; and 72°C for 8 min as the last elongation step. Fragment size analysis was conducted using GeneMarker (Softgenetics, State College, PA, USA) and then corrected using the FlexiBin Excel macro (Amos et al., 2010).

EST-SSR data were examined for typographic errors, scoring errors (e.g., allele drop-out, and stuttering), and estimates of null alleles with Micro-Checker (Van Oosterhout et al., 2004). Parameters of diversity were as follows: the proportion of distinguishable genotypes (PD) (Ellstrand and Roose, 1987) was measured as I (*G*/*N*), where G is the number of genets (distinct genotypes) and N is the total number of individuals sampled; for each population, the number of alleles, the percentage of polymorphic loci (*PPL*), *Nei*’s gene diversity (*Nei*), observed (*H*_o_), and expected (*H*_e_) heterozygosities were calculated using GenALEx 6.5.1 (Peakall and Smouse, 2006; Peakall and Smouse, 2012). The inbreeding coefficient (*F*_IS_) and allelic richness (*R*_s_) were calculated using FSTAT 2.9.3 (Goudet, 2001). All indices were adjusted for the frequency of null alleles (Van Oosterhout et al., 2004). An analysis of molecular variance (AMOVA) was performed, and the genetic differentiation index (*F*_ST_) was determined using Arlequin version 3.0, with significance tests based on 1,000 permutations (AMOVA, Excoffier et al., 2007).

### Common garden experiment

In the common garden experiment, we used the collected seeds of mother plants from the same populations in the wild to test the real growth and phenotypic differentiation among geographic populations and the response to genetic differentiation. For each of the nine geographic populations, we used the seeds from five collected mother plants within each of four populations. The experiment was carried out in a greenhouse at Huazhong Agricultural University, Wuhan, China (114°21’ E, 30°27’ N). Hubei has a subtropical monsoon climate, the average annual temperature is 16.4°C, and the average annual precipitation is approximately 1269 mm. From 17 December 2012 to 13 January 2013, we sowed the collected seeds in plastic trays (19.5 cm × 14.6 cm × 6.5 cm) filled with peat moss as substrate (Pindstrup Plus, Pindstrup Mossebrug A/S, Denmark). Because the time required for germination and the germination rate varied between the different populations, we sowed them on different dates to ensure that there were enough seedlings and that all the seedlings were in similar developmental stages at the start of the experiment. We placed all the trays with seeds in a greenhouse under natural light conditions, with a temperature between 20 and 26 °C.

On 25 February 2013, we transplanted similar-sized seedlings of each population into 5 L square plastic pots (24 cm × 24 cm × 15 cm) filled with field soil. The field soil included a total N of 0.78 ± 0.03 g kg^-1^, a total P of 0.58± 0.04 g kg^-1^, and a total K of 24.27 ± 1.15 g kg^-1^ (mean ± SE, n = 8). We transplanted one seedling of *E. annuus* in the center of each pot. After transplanting, we randomly assigned all pots in the greenhouse under natural light conditions, with a temperature between 20 and 28°C. We rerandomized the positions of all the pots every 4 weeks. There was a total of 180 pots (9 geographic populations × 4 populations × 5 mother plants = 180 pots).

On 12 June 2014 (i.e., fifteen weeks after transplanting, during the flowering period), we first calculated the plant height and then harvested the aboveground and underground biomass of the plants in all pots. Then, they were brought to the laboratory, separated into envelopes according to root (underground), stem, leaf, branch, and flower tissue, and dried at 80°C for 72 h in an oven. The total biomass of plants is the sum of aboveground biomass and underground biomass. The CV for each phenotypic trait was calculated.

### Data analysis

All statistical analyses were performed using R 4.0.3 (R Core Team, 2020). As geographic distances ranged from 30 km to 1425 km for the populations, the regions were defined as east (0-500 km), center (500-1000 km), or west (1000-1500 km). We then analyzed the effects of geographic region on growth traits (i.e., height, total biomass, flower biomass, leaf biomass, stem biomass, branch biomass, and root biomass) and their CV of *E. annuus* populations using a linear mixed-effects model with the ‘lme’ function in the ‘nlme’ package (Pinheiro et al., 2015). In these models, Region (east vs. center vs. west) was included as a fixed term. To account for the nonindependence of geographic populations (i.e., site) in a region, and populations within a geographic population, geographic population and population (nested within geographic populations) were included as random terms (Wang et al., 2019). We also included random structure to allow for variance between regions using the ‘varIdent’ function in the R package ‘nlm’ (Pinheiro et al., 2020). To meet the assumptions of normality of variance, plant height was sqrt-root transformed, flower biomass and total biomass were natural-log transformed, and the CV of growth was logit transformed (**Table 1**; **Supplementary Tables 3, 4**). In the linear mixed-effect models described above, we assessed the significance of fixed-effect-independent variables using likelihood-ratio tests (Zuur et al., 2009). A multiple post-hoc Tukey’s HSD test was used to compare the means for the phenotypic traits and their CV of *E. annuus* geographic populations.

**Table 1:** Statistical comparisons of genetic diversity indices for E. annuus in wild population. Distance, distance to the first recorded point of introduction; N, number of samples; Nei, Nei’ gene diversity; PPL, the percentage of polymorphic loci; H_o_, observed heterozygosity; H_e_, expected heterozygosity; F_IS_, inbreeding coefficient; R_s_, allelic richness and F_ST_, genetic differentiation index.

Region	Population	Distance	N	*Nei*	*PPL*	*H_o_*	*H_e_*	*F_IS_*	*R_s_*	*F_ST_*
		(km)			(%)					
East	JX	30	40	0.293	59.61	0.638	0.702	0.084	3.926	0.042
East	HZ	133	40	0.391	64.76	0.677	0.754	0.095	4.012	0.029
East	WZ	340	40	0.339	59.27	0.605	0.676	0.081	3.869	0.053
Center	XN	660	40	0.112	24.02	0.457	0.466	0.059	2.521	0.148
Center	WH	685	40	0.14	35.36	0.538	0.561	0.062	2.783	0.139
Center	XY	874	40	0.111	24.1	0.494	0.513	0.065	2.616	0.167
West	CK	1230	40	0.069	10.94	0.398	0.439	0.141	3.328	0.204
West	NC	1373	40	0.067	6.11	0.213	0.267	0.201	2.349	0.264
West	CQ	1425	40	0.068	8.14	0.312	0.368	0.117	2.483	0.235

Indices were calculated from allele frequencies of three microsatellite markers and adjusted for frequency of null alleles.

JX, Jiaxing; HZ, Hangzhou; WZ, Wenzhou; XN, Xianning; WH, Wuhan; XY, Xiangyang; CK, Chengkou; NC, Nanchuan; CQ, Chongqing.

Linear regression models were used to analyze the relationship between the growth of geographic populations in the wild experiment or in the common garden experiment and the genetic diversity or variation of geographic populations in the wild experiment, and geographic distance to the FRPI. We also analyzed the correlation between key traits (height, total biomass, and flower biomass) of geographic populations in the wild and their genetic diversity. We used ANCOVA to compare the difference between slopes of the linear regression between the growth of the wild experiment or of the common garden experiment and geographic distance to the FRPI.

## Results

### Growth and phenotype of *E. annuus* populations in wild and common garden experiments with geographic distance

**Figure 1:**
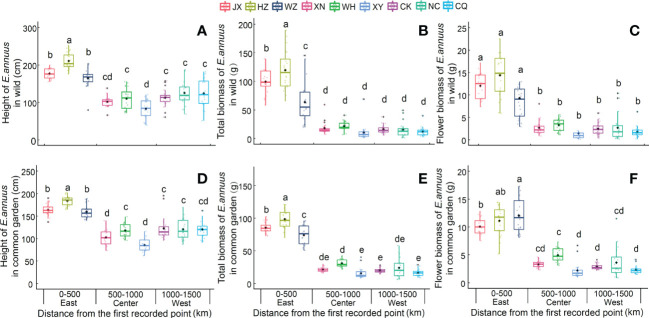
Height **(A, D)**, total biomass **(B, E)** and flower biomass **(C, F)** of E. annuus geographic populations in different regions in wild and common garden experiments. Mean ± SE were given. Different lowercase letters mean statistically significant differences (P < 0.05) JX, Jiaxing; HZ, Hangzhou; WZ, Wenzhou; XN, Xianning; WH, Wuhan, XY, Xiangyang; CK, Chengkou; NC, Nanchuan; CQ, Chongqing.

The plant height and biomass of the *E. annuus* populations in both the wild and common garden experiments exhibited great differences between the three regions in China (**Table 2**). Specifically, height, total biomass, and flower biomass in the eastern region (i.e., short geographic distance to the FRPI and long-term introduced) were significantly greater than in the center and western (i.e., long geographic distance to FRPI, and short-term introduced) regions (**Figure 1**). The plant phenotype also exhibited a clump ecotype in the eastern region and a scatter ecotype in the central and western regions in both the wild and common garden experiments. Height and total biomass among geographic populations in the eastern region also varied greatly, with the greatest values in the Hangzhou (HZ) population and the lowest values in the Wenzhou (WZ) population (**Figure 1**). Leaf biomass, stem biomass, branch biomass, and root biomass showed similar patterns to plant height and total biomass **(Supplementary Table 3**; **Supplementary Figure 2**). Furthermore, the height, total biomass, and flower biomass of *E. annuus* populations in both the wild and common garden experiments decreased with geographic distance to the FRPI (**Supplementary Figure 4**), indicating the trait advantage of long-term introduced populations to invasion success. However, their negative correlations were steeper under wild conditions than under common garden conditions (slopes of the linear regression between the two experiments were x and y: height, ANCOVA, *F* = 2.474, *P* = 0.013; total biomass, ANCOVA, *F* = 4.731, *P* < 0.001; flower biomass, ANCOVA, *F* = 5.337, *P* < 0.001).

**Table 2:** Results of linear mixed-effects models testing the effects of region (East vs. Center vs. West) on height, total biomass, flower biomass in wild or common garden experiments in China.

Wild experiment		Height (square root transformed)		Total biomass (square root transformed)		Flower biomass (square root transformed)	
***Fixed effects***	df	*X* ^2^	*p*	*X* ^2^	*p*	*X* ^2^	*p*
Region	2	**18.82**	**<0.001**	**20.72**	**<0.001**	**22.49**	**<0.001**
***Random effects***		SD		SD		SD	
Geographic population		0.55		0.79		0.27	
population		0.73		0.76		0.34	
Residual		0.72		0.66		0.43	
		*R* ^2^m	*R* ^2^c	*R* ^2^m	*R* ^2^c	*R* ^2^m	*R* ^2^c
*R* ^2^ of the model		0.63	0.86	0.73	0.89	0.69	0.85
Common garden experiment		Height (square root transformed)		Total biomass (log root transformed)		Flower biomass (log root transformed)	
***Fixed effects***	df	*X* ^2^	*p*	*X* ^2^	*p*	*X* ^2^	*p*
Region	2	**19.45**	**<0.001**	**22.65**	**<0.001**	**19.01**	**<0.001**
***Random effects***		SD		SD		SD	
Geographic population		0.48		0.2		0.24	
*p*opulation		0.38		0.29		0.31	
Residual		0.53		0.2		0.21	
		*R* ^2^m	*R* ^2^c	*R* ^2^m	*R* ^2^c	*R* ^2^m	*R* ^2^c
*R* ^2^ of the model		0.69	0.87	0.75	0.94	0.67	0.92

R^2^m: Marginal R^2^; R^2^c: Conditional R^2^; Significant effects (p <0.05) are in bold.

### Coefficient of variation of growth of *E. annuus* populations in the common garden experiment

**Figure 2:**
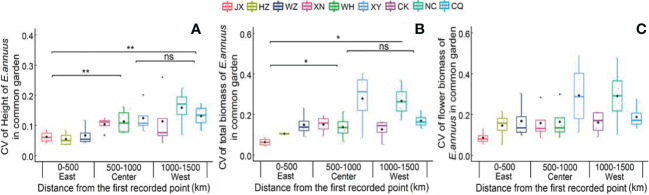
Coefficient of variation (CV) of height **(A)**, total biomass **(B)** and flower biomass **(C)** of E. annuus geographic populations of common garden experiments in different regions. Mean ± SE were given. The significance levels are: **p < 0.01, *p < 0.05, and ns, p > 0.05. JX, Jiaxing; HZ, Hangzhou; WZ, Wenzhou; N, Xianning; WH, Wuhan; XY, Xiangyang; CK, Chengkou; NC, Nanchuan; CQ, Chongqing.

In the common garden experiment, the CVs of height and total biomass were lowest in the eastern region, showing great stability in growth traits in the long-term introduced populations (**Figure 2**). Moreover, the CV of flower biomass was not different between the three different regions in the common garden experiment, indicating a low variation range of reproductive traits between the different populations.

### Genetic diversity or variation in wild *E. annuus* populations with geographic distance

**Figure 3:**
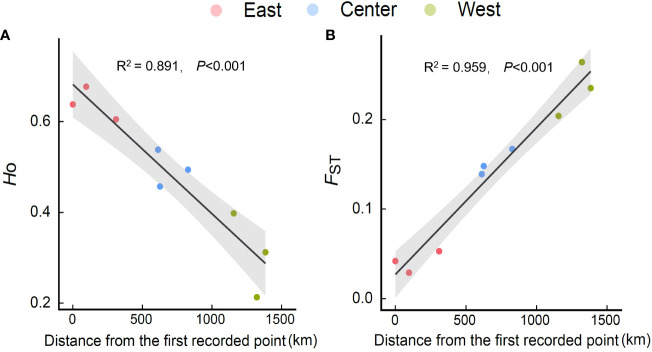
Relationships of H_o_ [**(A)** observed heterozygosity] and F_ST_ [**(B)** fixation index] of *E. annuus* geographic populations with the distance to the first recorded point of introduction (FRPI). Similar symbols are different sites within the same region.

Within regions, the percentage of polymorphic loci (*PPL*), Nei’s gene diversity (*Nei*), the proportion of distinguishable genotypes (*G/N*), observed heterozygosity (*H*_o_), expected heterozygosity (*H*_e_), and allelic richness (*R*_s_) were higher in the eastern region and lowest in the western region (especially in Hangzhou [HZ] and Nanchuan (NC)) (**Table 1**; **Supplementary Table 5**). The *H*_o_, *H_e_*, and *R*_s_ of *E. annuus* wild populations were significantly negatively related to geographic distance to the FRPI (**Figure 3A**; **Supplementary Figures 5A-C**). These results indicate that genetic diversity decreased from long-term to short-term introduced wild populations. The genetic differentiation index (*F*_ST_) and inbreeding coefficient (*F*_IS_) were significantly positively related to geographic distance to the FRPI (**Table 1**; **Figure 3B**; **Supplementary Figure 5D**). AMOVA showed a definite geographic trend of genetic differentiation in *E. annuus* populations in China, with 23% variation between regions, 38% between populations, and 39% within populations (**Table 3**; **Figure 3**).

**Table 3:** Analysis of molecular variance (AMOVA) results of *E. annuus* populations based on microsatellite markers.

Source of variation	DF	Sum of squares	Variance component	Percentage of variation (%)	*P* value
Among regions	2	347.12	166.28	23	**<0.01**
Among populations	33	1987.45	67.59	38	**<0.01**
Within populations	324	1083.22	4.87	39	**<0.01**

Significant effects (p <0.05) are in bold.

### Relationships between the growth and genetic variation of *E. annuus* populations

**Figure 4:**
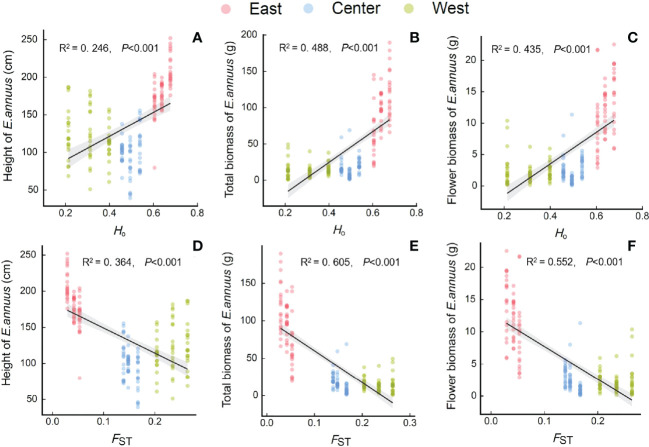
Relationships between genetic diversity and differentiation [**(A-C)** H_o_, observed heterozygosity; **(D-F)**, F_ST_, genetic differentiation index] with height **(A, D)**, total biomass **(B, E)** and flower biomass **(C, F)** of *E. annuus* geographic populations in different regions in China. Similar symbols are different sites within the same region.

The height, total biomass, and flower biomass of *E. annuus* populations were significantly positively correlated with *H*_o_ (**Figures 4A–C**), and significantly negatively correlated with *F*_ST_ (**Figures 4D–F**). Furthermore, greater height and biomass were associated with higher *H*_o_ or lower *F*_ST_ in the eastern populations than in the central and western populations.

## Discussion

We found that *E. annuus* populations had better growth performance (i.e., height and biomass) and genetic diversity, and less variation of traits and genetic differentiation in the long-term introduced region (i.e., east) than in the short-term introduced (west) region in China. Furthermore, within populations, genetic diversity showed a similar pattern to growth performance, which indicated significantly positive or negative correlations between growth performance and genetic diversity or genetic differentiation, especially for common garden experiments. These results indicate that growth traits and genetic variation of populations covary in response to regional adaptation and spread, and populations that entered earlier have parallel high genetic diversity and high growth dominance in introduced environments.

### Growth and phenotypic differentiation in *E. annuus* populations

Our results showed that in *E. annuus* populations within wild and common garden conditions, growth traits were significantly more varied, whereas their CVs were significantly lower in the eastern region than in the central and western regions, indicating a decrease in growth and an increase in phenotypic differentiation with invasion time. The *E. annuus* populations had a better growth advantage in the long-term introduced region. This is consistent with previous findings that populations of some invasive plant species in the earlier introduced ranges grew taller and produced larger biomass than those in the later ranges (Sultan et al., 2013; Jeschke and Heger, 2018; Egbon et al., 2020; Querns et al., 2022). Although previous report demonstrated that plant growth and functional traits are affected by soil nutrients and spatial gradients in the field (Wang et al., 2017), our results from common garden experiments showed convincing findings similar to those of wild experiments, indicating that growth and phenotypic differentiation are probably related to the genetic variation of *E. annuus*. A potential mechanism is likely to be that invasive plants have greater competitive traits under new abiotic and biotic environments after being introduced into a new area (Sultan, 1995; Prentis et al., 2008; Sultan et al., 2013; Jeschke and Heger, 2018). We also found that *E. annuus* exhibited both clump and scatter ecotypes (but clump dominated) in the eastern region, and only the scatter ecotype in the western region, in both the wild and common garden experiments. The clump ecotype had more branches and total biomass, which may be an adaptive advantage to interferences in farmland habitats (i.e., herbicide, herbivory, and trampling) through risk sharing between multiple branches (our experimental observation). This is supported by experimental evidence showing that dominant ecotypes of *Imperata cylindrica* can be maintained in the early stages of invasion and can invade a wider area with more dominant traits (common ecotype [C-type] and early flowering ecotype [E-type] are found scattered in the Japanese Islands) (Matumura and Yukimura, 1980; Maeda et al., 2009). Similarly, dominant ecotypes (hexaploid and octoploid) of *Fallopia sachalinensis* prevail across the invaded area but not in the native range (Mandák et al., 2003). In our study, the high consistency of growth and phenotype of *E. annuus* populations in long-term (different geographical distance gradients) introduced regions in wild and common garden conditions indicated largely genetic effects. This result is in line with the variable performance of many invasive plants (*Mimulus guttatus*, *Polygonum cespitosum*, *Triadica sebifera*, and *Catorhintha schaffneri*) in the regions they were introduced, indicating that adaptive evolution of invasive plants occurs in the new region (Kawecki and Ebert, 2004; Huang et al., 2013; Sultan et al., 2013; Huang et al., 2015; Egbon et al., 2020; Querns et al., 2022).

### Genetic diversity and genetic differentiation in *E. annuus* populations

We found that the genetic diversity of *E. annuus* significantly decreased with geographic distance, with the same pattern as growth, i.e., highest in the long-term introduced region and lowest in the short-term introduction region. The rapid genetic decline of *E. annuus* with geographic distance to the FRPI is consistent with previous studies showing that alien species may decrease their genetic diversity during the invasion process and maintain population genetics depending on changes in selection, genetic drift, and gene flow (Broennimann et al., 2012; Dlugosch et al., 2015). Ohadi et al. (2016) found high rates of gene variation among *Cakile maritima* populations occupying long-term invaded regions. Moreover, genetic diversity was significantly positively correlated with height and biomass in *E. annuus* with geographic distance, suggesting that long-term introduced populations had higher genetic diversity and variation, as well as parallelly higher growth adaptability and higher vitality than short-term introduced populations. Previous studies have shown that genetic diversity and variation in *E. annuus* may reflect a variety of genotype frequencies and apomictic reproduction in long-distance dispersal (Ellstrand and Roose, 1987; Edwards et al., 2006; Trtikova et al., 2011). Apomixis is typically a reproductive advantage for *E. annuus*, allowing better preservation of the dominant traits (i.e., height and biomass) of the mother plant and more successful spread, but reducing genetic diversity. Thus, *E. annuus* can maintain the dominant performance of the mother plant in the offspring. Therefore, eastern geographic populations had more dominant genotypes, i.e., clump ecotype, which have been well preserved for a long time (Trtikova et al., 2011; Sultan et al., 2013; Querns et al., 2022). However, there were fewer dominant genotypes in the western region due to increasing gene diversity. Furthermore, the CVs of height, total biomass, and flower biomass in wild geographical populations were also higher in the western region, suggesting that relatively short-term introduced populations had greater variation and instability of growth performance, and were more influenced by regional environments. In our common garden experiment, the CV of growth traits was generally consistent with the wild experiments influenced by environmental conditions. From our common garden experiment, the transgeneration presented long-term environmental adaptation and characterized the variation of plant traits with genetic diversity and variation (Depardieu et al., 2020). The parallel high variability in growth traits and high genetic differentiation among western geographical populations is likely to depend on the limitation of seed dispersal and adaptive time. In contrast, eastern geographical populations had high levels of genotypes based on high gene diversity in long-term evolution. These genotypes also exhibited similar growth dominance, consistent with the lowest growth variation and high stability. Therefore, the consistency of high genetic diversity and high growth dominance in *E. annuus* populations have allowed them to easily invade the eastern range.

### Coefficient of variation in common garden conditions

Although we found a greater decrease in growth with geographic distance in wild conditions than in common garden conditions, there was no significant environmental effect on population growth, suggesting that the different growth performances of *E. annuus* populations with geographical distance were attributed to genetic variation. Similarly, previous studies showed that variation in the growth fitness of *E. annuus* did not depend on the patterns of environmental variation (Stratton, 1992; Stratton, 1994). However, we observed that the CV of flower biomass between different regions was not different under common garden conditions but under wild conditions. This may have resulted from environmental changes (i.e., availability resources and stress) rather than genetic variation, which is in line with the finding that reproductive (i.e., flower biomass) fitness in the invasive plant *Polygonum cespitosum* altered in its introduced range (Alpert and Simms, 2002; Richardson and Pyšek, 2006; Sultan et al., 2013; Sultan and Matesanz, 2015). Additionally, the success of invasive grass *Pennisetum setaceum* populations in their invasive ranges resulted in high environmental adaptation to reproduction (Williams et al., 1995; Poulin et al., 2005; Poulin et al., 2007). On the other hand, the different growth and reproduction patterns of *E. annuus* in long- or short-term introduced regions might result from naturally selected dominant genotypes and genetic variation. *E. annuus* is an apomictic species in which nondominant genotypes are eliminated in the long-distance spread process (Noyes, 2000; Trtikova et al., 2010; Trtikova et al., 2011). Moreover, the reproductive stage of *E. annuus* might provide more high-quality seed production and the generation of new genotypes under different environmental conditions (Baker, 1965; Noyes, 2000; Trtikova et al., 2011).

Our experiment did not test the distinguishing genotypes among populations, and it would be difficult to translate the results to evaluate genotype differences with geographic distance. Additionally, the correlations between growth traits and genetic variation at the population level might not demonstrate evolutionary adaptation for *E. annuus.* However, the integration of wild and common garden experiments can largely reveal genotypic differences between different regions in China.

## Conclusions

Our results indicate that the growth traits and genetic variation of *E. annuus* populations covary in response to adaptation and spread, and populations that entered introduced regions earlier have consistently high genetic diversity and high growth dominance. However, the short-term nature of our experiments with the population of *E. annuus* meant that our investigation was somewhat limited. Therefore, longer-term field research is needed to test whether different performances among populations occur at different geographical distances. Future studies should also test the genetic structure and invasion history at broader geographic distances in China or worldwide. However, we conclude that parallel genetic and phenotypic variation with invaded geographical distance, growth, and reproductive traits can be used as important predictors of the adaptation and genetic variation of invasive plants.

## Data availability statement

The datasets presented in this study can be found in online repositories. The names of the repository/repositories and accession number(s) can be found in the article/**Supplementary Material**.

## Author contributions

Y-JW set up the experiment. Y-JW, X-PS and ZL conducted field sampling. X-PS and Y-YL conducted molecular analysis. Q-FY and Y-YL carry out the statistical analysis. Y-YL. wrote the first draft of the manuscript. Y-JW, Z-XZ, X-PS and Y-YL contributed substantially to the revisions. All authors contributed to the article and approved the submitted version.
