# Supplementary material for: Parallel genetic and phenotypic differentiation of Erigeron annuus invasion in China
Source: Front Plant Sci. 2023 Jan 4;13:994367. doi: 10.3389/fpls.2022.994367 (PMC9845934; doi:10.3389/fpls.2022.994367)
Supplement: Supplementary file 1 [file DataSheet_1.docx]

**Supplement materials**

**Supplementary Table 1.** Environmental features of *E. annuus* geographic populations in different regions in China.

| Region | City | Slope (°) | Longitude | Latitude | Average annual temperature(^o^C) | Average annual rainfall(mm) | Habitats |
| --- | --- | --- | --- | --- | --- | --- | --- |
| East | Wenzhou | 0-1 | 121°25' | 28°39' | 17.5 | 1650 | Farmlands |
| East | Hangzhou | 0-1 | 120°28' | 28°62' | 17.8 | 1454 | Farmlands |
| East | Jiaxing | 0-2 | 121°37' | 29°51' | 16.4 | 1193 | Farmlands |
| Center | Xianning | 0-2 | 114°21' | 30°28' | 16.5 | 1530 | Farmlands |
| Center | Wuhan | 0.2 | 114°21' | 30°27' | 16.4 | 1269 | Farmlands |
| Center | Xiangyang | 0-2 | 114°23' | 31°05' | 16.4 | 1020 | Farmlands |
| West | Nanchuan | 2-5 | 107°11' | 29°10' | 11.3 | 1100 | Farmlands |
| West | Chongqing | 2-4 | 106°57' | 29°30' | 16.0 | 1175 | Farmlands |
| West | Chenkou | 2-3 | 106°23' | 29°50' | 14.6 | 1319 | Farmlands |

<http://data.cma.cn>

**Supplementary Table 2.** Characteristic of three microsatellite primers (polymorphic EST-SSR) in *E. annuus*. Shown for each primer pair were the forward (F) and reverse (R) primer sequences, repeat type, size range of amplification fragment, annealing temperature (Ta), GenBank accession number.

| Locus | Primer sequence (5’-3’) | Repeat motif | Size range (bp) | Ta (◦C) | Genbank No. |
| --- | --- | --- | --- | --- | --- |
| SSR-01 | F:GTGGCAGAAGATGGAGTAAT  R:ATCATCATCATCACCACCAC | (GAT)18 | 201 | 55.3 | BQ864089 |
| SSR-02 | F:CCTCCATAACCAAAACCTCA  R:ACTTCTTTCGACATGCTTCT | (AG)23 | 128 | 55 | DW130931 |
| SSR-03 | F:CAGATCAAGCGGGTAACTAA  R:TATCAAGACCCTAAGCGAAC | (TC)25 | 152 | 56.8 | DW127456 |

**Supplementary Table 3.** Results of linear mixed-effects models testing the effects of region (East vs. Center vs. West) on leaf biomass, stem biomass, branch biomass and root biomass of *E. annuus* geographic populations of wild and common garden experiments in China.

| **Wild experiment** | |  | Leaf biomass  (log transformed) | | | | | | Stem biomass (log transformed) | | | | | Branch biomass (cubic transformed) | | | | | | | Root biomass  (cubic transformed) | | | | | | |
| --- | --- | --- | --- | --- | --- | --- | --- | --- | --- | --- | --- | --- | --- | --- | --- | --- | --- | --- | --- | --- | --- | --- | --- | --- | --- | --- | --- |
| **Fixed effect** | | df | χ^2^ | | | *p* | | χ^2^ | | | *p* | | χ^2^ | | | | *p* | | | χ^2^ | | | | *p* | | | |
| Region | | 2 | **20.13** | | | **<0.001** | | **14.83** | | | **0.001** | | **23.08** | | | | **<0.001** | | | **24.39** | | | | **<0.001** | | | |
| **Random effect** | |  | SD | | |  | | SD | | |  | | SD | | | |  | | | SD | | | |  | | | |
| Geographic population | |  | 0.36 | | |  | | 0.45 | | |  | | 0.14 | | | |  | | | 0.11 | | | |  | | | |
| Population | |  | 0.36 | | |  | | 0.39 | | |  | | 0.25 | | | |  | | | 0.16 | | | |  | | | |
| Residual | |  | 0.42 | | |  | | 0.44 | | |  | | 0.27 | | | |  | | | 0.17 | | | |  | | | |
|  | |  | R^2^m | | | R^2^c | | R^2^m | | | R^2^c | | R^2^m | | | | R^2^c | | | R^2^m | | | | R^2^c | | | |
| R^2^ of the model | |  | 0.70 | | | 0.88 | | 0.57 | | | 0.85 | | 0.69 | | | | 0.85 | | | 0.74 | | | | 0.88 | | | |
|  |  | | |  |  | |  | | |  | | | | |  |  | | |  | | |  |  | | |  |  |
| **Common garden experiment** | |  | Leaf biomass (log transformed) | | | | | | Stem biomass (log transformed) | | | | | Branch biomass (log transformed) | | | | | | | Root biomass (sqrt root transformed) | | | | | | |
| **Fixed effect** | | df | χ^2^ | | | *p* | | | χ^2^ | | | *p* | | χ^2^ | | | | *p* | | | χ^2^ | | | | *p* | | |
| Region | | **2** | **24.6332** | | | **<0.001** | | | **17.31** | | | **<0.001** | | **27.45** | | | | **<0.001** | | | **27.71** | | | | **<0.001** | | |
| **Random effect** | |  | SD | | |  | | | SD | | |  | | SD | | | |  | | | SD | | | |  | | |
| Geographic population | |  | 0.17 | | |  | | | 0.29 | | |  | | 0.15 | | | |  | | | 0.15 | | | |  | | |
| Population | |  | 0.40 | | |  | | | 0.30 | | |  | | 0.30 | | | |  | | | 0.18 | | | |  | | |
| Residual | |  | 0.18 | | |  | | | 0.21 | | |  | | 0.25 | | | |  | | | 0.14 | | | |  | | |
|  | |  | R^2^m | | | R^2^c | | | R^2^m | | | R^2^c | | R^2^m | | | | R^2^c | | | R^2^m | | | | R^2^c | | |
| R^2^ of the model | |  | 0.76 | | | 0.96 | | | 0.66 | | | 0.93 | | 0.79 | | | | 0.93 | | | 0.86 | | | | 0.97 | | |

R^2^m: Marginal R^2^; R^2^c: Conditional R^2^; Significant effects (*p* <0.05) are in bold.**Supplementary Table 4.** Results of linear mixed-effects models testing the effects of region (East vs. Center vs. West) on coefficient of variation (CV) of growth and reproduction traits of *E. annuus* geographic population of common garden experiments in China.

| **Common garden experiment** |  | CV of height (logit transformed) | | | CV of total biomass (logit transformed) | | | | | CV of flower biomass (log transformed) | | | | |  | | | |
| --- | --- | --- | --- | --- | --- | --- | --- | --- | --- | --- | --- | --- | --- | --- | --- | --- | --- | --- |
| **Fixed effect** | df | χ^2^ | | *p* | χ^2^ | | | *p* | | χ^2^ | | | *p* | |  | | |  |
| Region | 2 | **11.39** | | **0.003** | 4.19 | | | 0.12 | | **6.72** | | | **0.035** | |  | | |  |
| **Random** |  | SD | |  | SD | | |  | | SD | | |  | |  | | |  |
| Geographic population |  | 0.00 | |  | 0.58 | | |  | | 0.11 | | |  | |  | | |  |
| Population |  | 0.39 | |  | 0.00 | | |  | | 0.14 | | |  | |  | | |  |
| Residual |  | 0.32 | |  | 0.00 | | |  | | 0.36 | | |  | |  | | |  |
|  |  | R^2^m | | R^2^c | R^2^m | | | R^2^c | | R^2^m | | | R^2^c | |  | | |  |
| R^2^ of the model |  | 0.36 | | 0.74 | 0.29 | | | 0.98 | | 0.36 | | | 0.66 | |  | | |  |
|  |  |  |  | | | |  | |  | | |  | |  | | |  |  |
| **Common garden experiment** |  | CV of leaf biomass (cubic transformed) | | | | CV of stem biomass (log transformed) | | | | | CV of branch biomass (log transformed) | | | | | CV of root biomass (log transformed) | | |
| **Fixed effect** | df | χ^2^ | | *p* | χ^2^ | | | *p* | | χ^2^ | | | *p* | | χ^2^ | | | *p* |
| Region | 2 | **4.92** | | ***0.08*** | **11.84** | | | **0.003** | | 2.90 | | | 0.23 | | **7.62** | | | **0.02** |
| **Random effect** |  | SD | |  | SD | | |  | | SD | | |  | | SD | | |  |
| Geographic population |  | 0.08 | |  | 0.24 | | |  | | 0.17 | | |  | | 0.29 | | |  |
| Population |  | 0.00 | |  | 0.27 | | |  | | 0.27 | | |  | | 0.24 | | |  |
| Residual |  | 0.00 | |  | 0.19 | | |  | | 0.29 | | |  | | 0.21 | | |  |
|  |  | R^2^m | | R^2^c | R^2^m | | | R^2^c | | R^2^m | | | R^2^c | | R^2^m | | | R^2^c |
| R^2^ of the model |  | 0.33 | | 0.98 | 0.50 | | | 0.89 | | 0.08 | | | 0.60 | | 0.35 | | | 0.84 |

R^2^m: Marginal R^2^; R^2^c: Conditional R^2^; Significant effects (*p* <0.05) are in bold, while marginally significant effects (0.05<*p*<0.1) are underlined and in bold.

**Supplementary Table 5.** Genotypes of *E. annuus* found in different regions in China. N, number of samples; G, number of detected genotypes; I (G/N), proportion of distinguishable genotypes. JX: Jiaxing; HZ: Hangzhou; WZ: Wenzhou; XN: Xianning; WH: Wuhan, XY: Xiangyang; CK: Chengkou; NC: Nanchuan; CQ: Chongqing.

| Region | Population | N | G | I  (G/N) |
| --- | --- | --- | --- | --- |
| East | HZ1 | 10 | 3 | 0.30 |
| East | HZ2 | 10 | 2 | 0.20 |
| East | HZ3 | 10 | 4 | 0.40 |
| East | HZ4 | 10 | 3 | 0.30 |
| East | JX1 | 10 | 3 | 0.30 |
| East | JX2 | 10 | 2 | 0.20 |
| East | JX3 | 10 | 2 | 0.20 |
| East | JX4 | 10 | 3 | 0.30 |
| East | WZ1 | 10 | 2 | 0.20 |
| East | WZ2 | 10 | 3 | 0.30 |
| East | WZ3 | 10 | 3 | 0.30 |
| East | WZ4 | 10 | 3 | 0.30 |
| Center | WH1 | 10 | 2 | 0.20 |
| Center | WH2 | 10 | 1 | 0.10 |
| Center | WH3 | 10 | 2 | 0.20 |
| Center | WH4 | 10 | 2 | 0.20 |
| Center | XY1 | 10 | 2 | 0.20 |
| Center | XY2 | 10 | 1 | 0.10 |
| Center | XY3 | 10 | 1 | 0.10 |
| Center | XY4 | 10 | 2 | 0.20 |
| Center | XN1 | 10 | 2 | 0.20 |
| Center | XN2 | 10 | 2 | 0.20 |
| Center | XN3 | 10 | 1 | 0.10 |
| Center | XN4 | 10 | 1 | 0.10 |
| West | CK1 | 10 | 1 | 0.10 |
| West | CK2 | 10 | 2 | 0.20 |
| West | CK3 | 10 | 1 | 0.10 |
| West | CK4 | 10 | 1 | 0.10 |
| West | CQ1 | 10 | 2 | 0.20 |
| West | CQ2 | 10 | 1 | 0.10 |
| West | CQ3 | 10 | 1 | 0.10 |
| West | CQ4 | 10 | 1 | 0.10 |
| West | NC1 | 10 | 1 | 0.10 |
| West | NC2 | 10 | 1 | 0.10 |
| West | NC3 | 10 | 2 | 0.20 |
| West | NC4 | 10 | 1 | 0.10 |


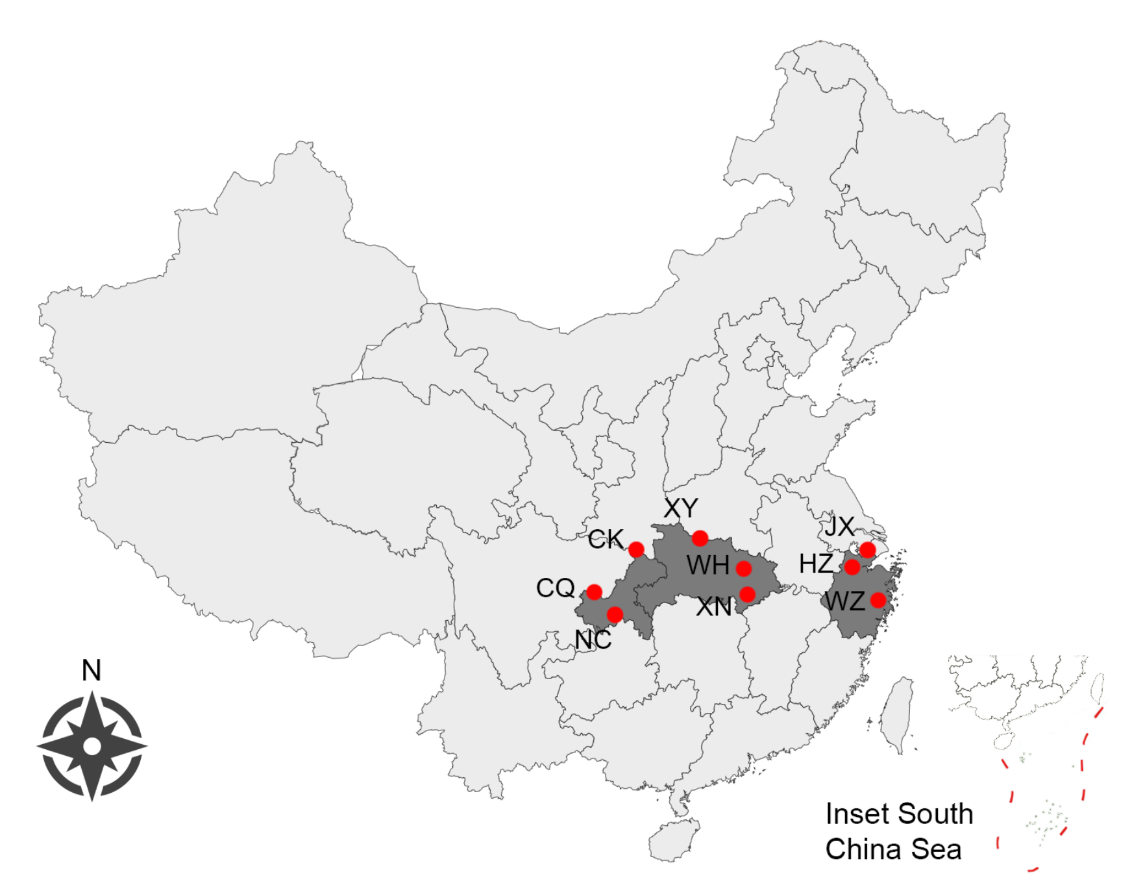
**Supplementary Figure 1.** Geographic populations of *E. annuus* in different regions in China. JX: Jiaxing; HZ: Hangzhou; WZ: Wenzhou; XN: Xianning; WH: Wuhan, XY: Xiangyang; CK: Chengkou; NC: Nanchuan; CQ: Chongqing.

**Supplementary Figure 2.** Leaf biomass (A and E), stem biomass (B and F), branch biomass (C and G) and root biomass (D and H) of *E. annuus* geographic populations of wild and common garden experiments in China. Mean ± SE were given. JX: Jiaxing; HZ: Hangzhou; WZ: Wenzhou; XN: Xianning; WH: Wuhan, XY: Xiangyang; CK: Chengkou; NC: Nanchuan; CQ: Chongqing.


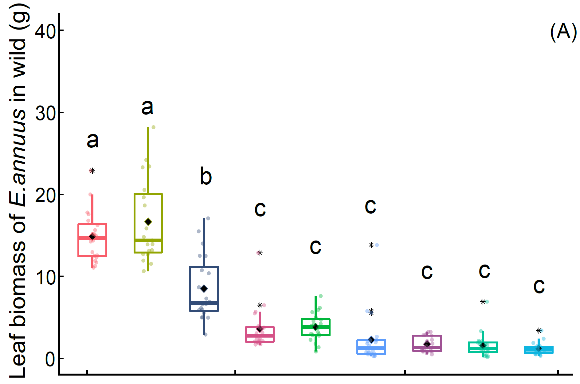

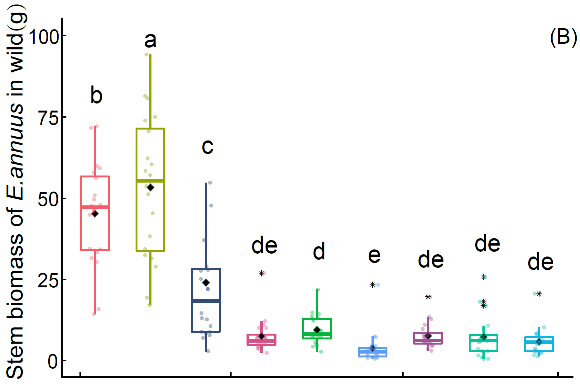

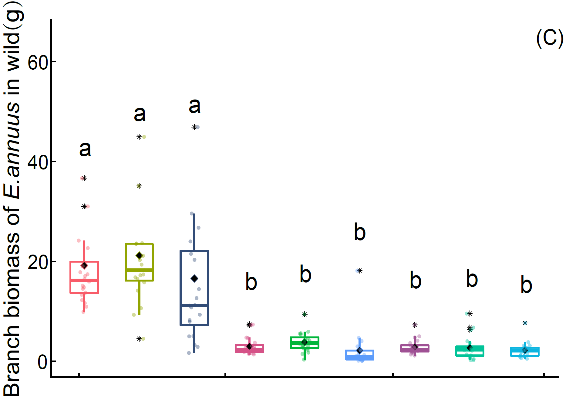

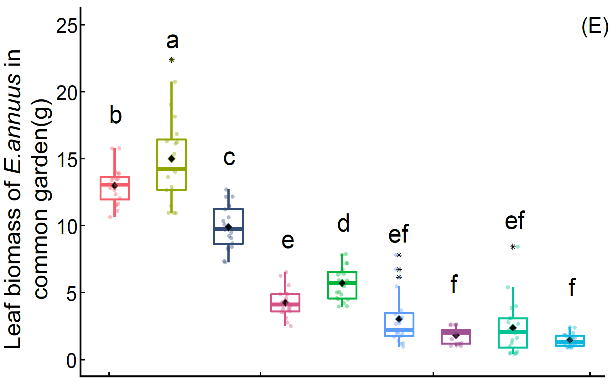

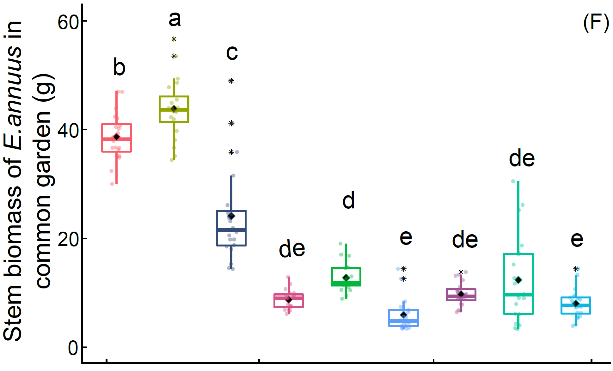

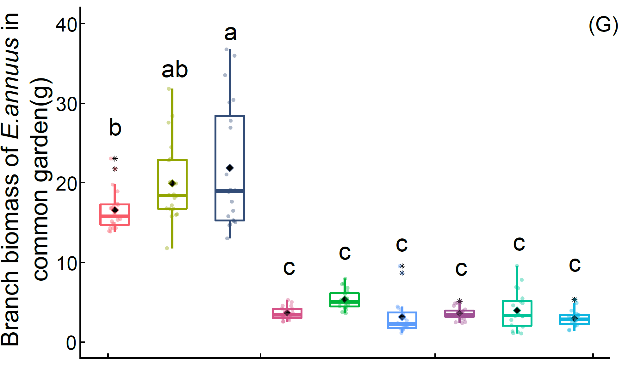

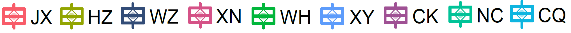

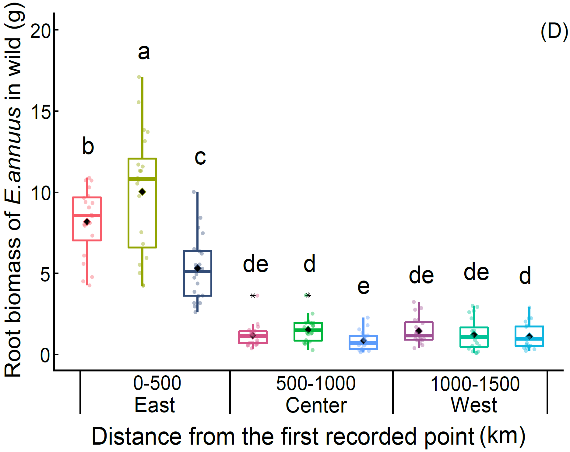

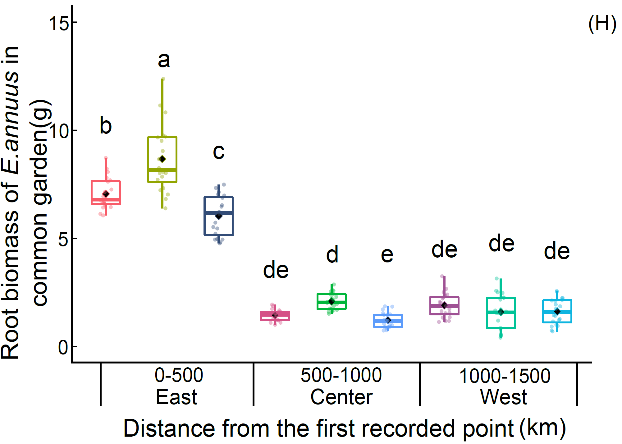


**
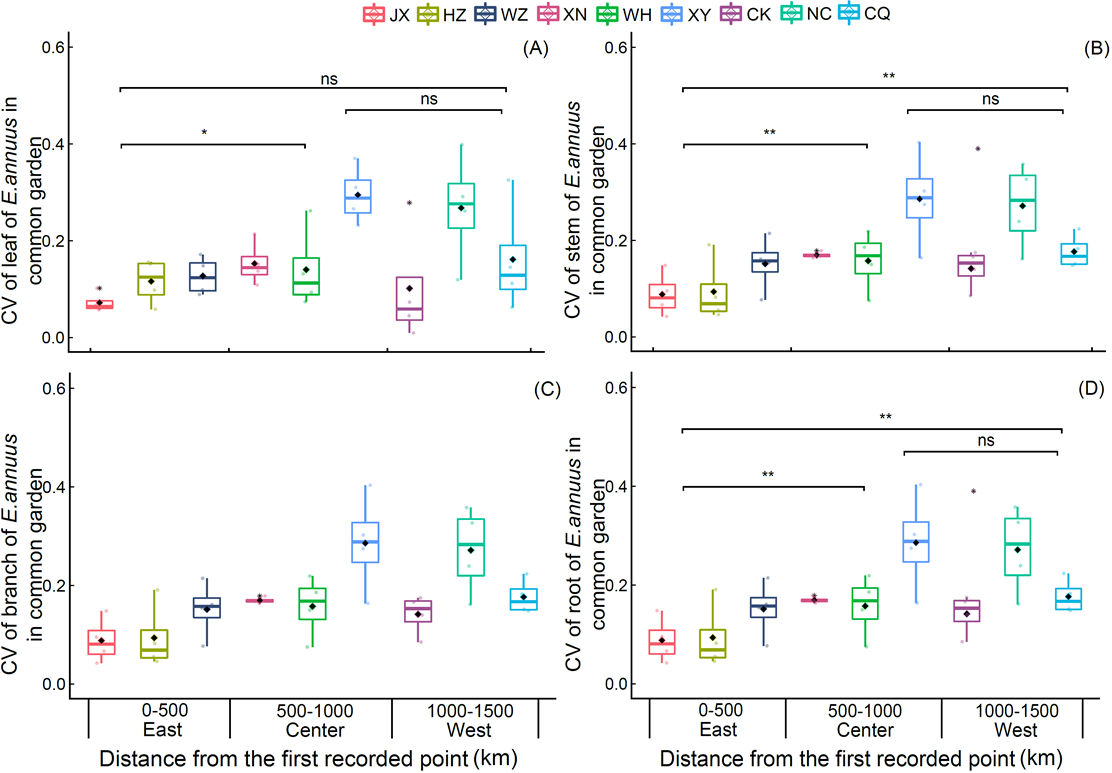
**

**Supplementary Figure 3.** Coefficient of variation (CV) of leaf biomass (A), stem biomass (B), branch biomass (C) and root biomass (D) of *E. annuus* geographic populations of common garden experiments in China. Mean ± SE were given. JX: Jiaxing; HZ: Hangzhou; WZ: Wenzhou; XN: Xianning; WH: Wuhan, XY: Xiangyang; CK: Chengkou; NC: Nanchuan; CQ: Chongqing.


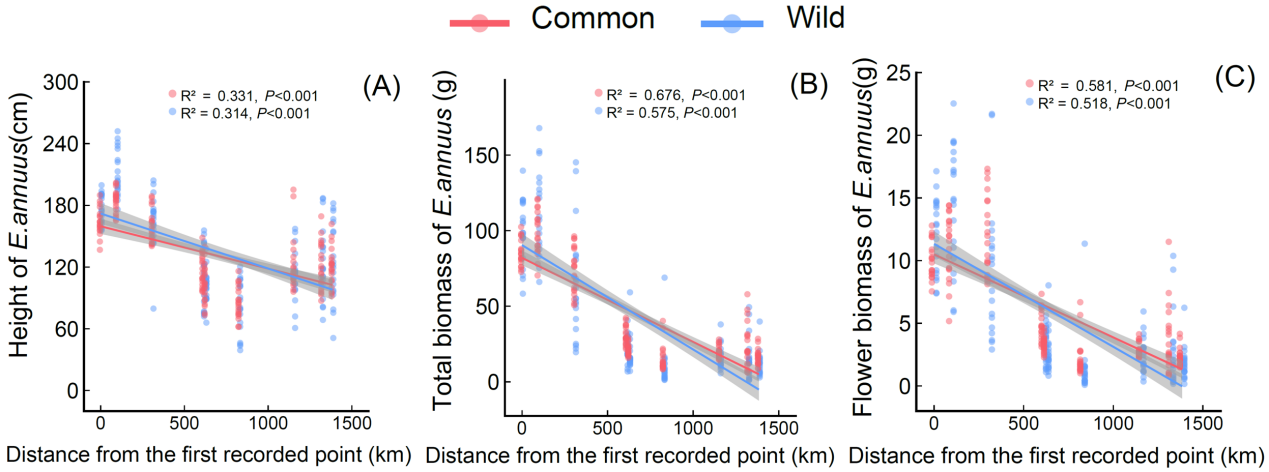
**Supplementary Figure 4.** Relationships of height (A), total biomass (B) and flower biomass (C) of *E. annuus* geographic populations in wild and common garden experiments with the distance to the first recorded point of introduction (FRPI).
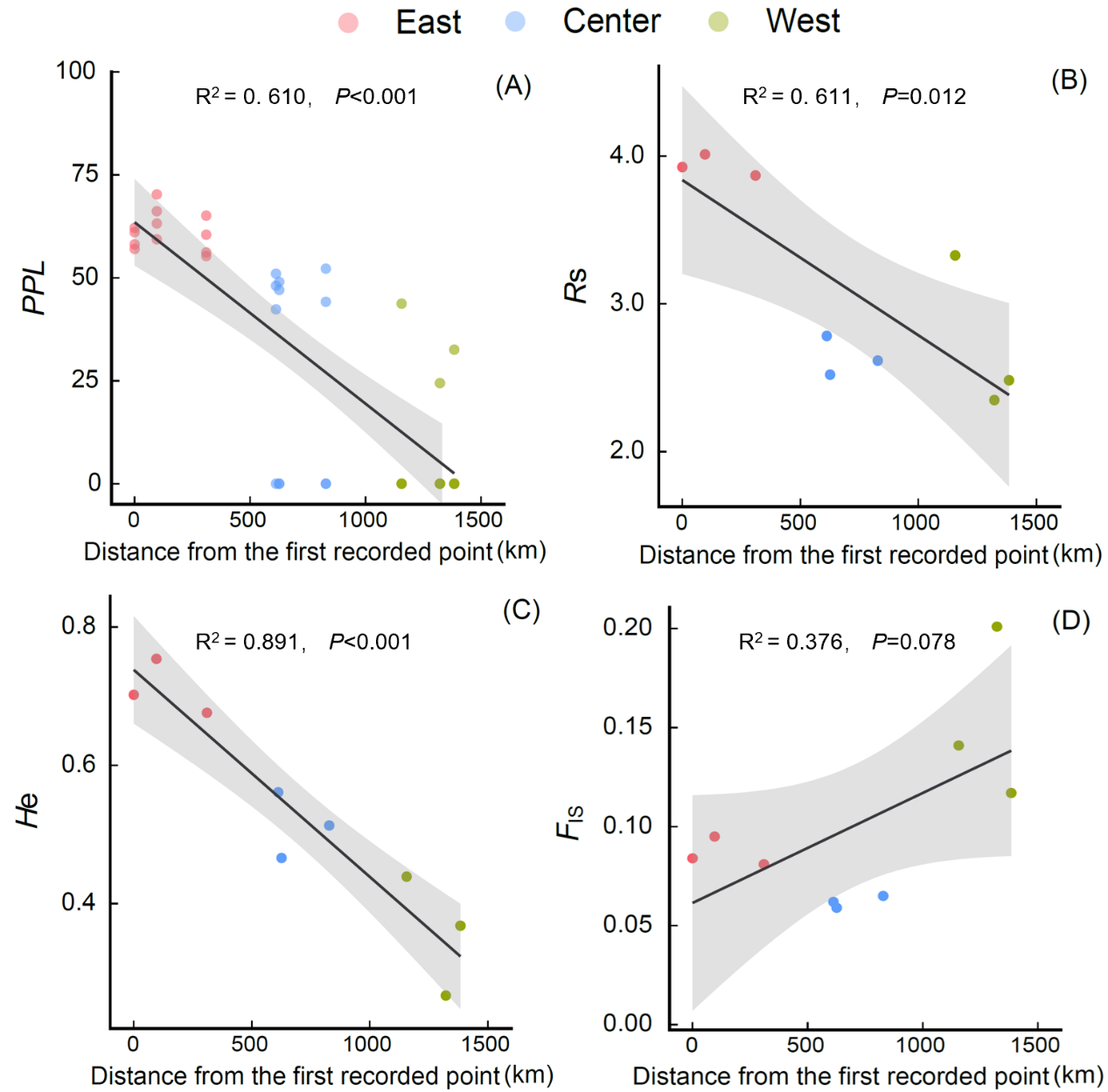
**Supplementary Figure 5.** Relationships of population genetic parameters of *E. annuus* with the distance from the first recorded point of introduction (FRPI). Similar symbols are different sites within the same region. (*PPL*, the percentage of polymorphic loci; *R*_s_, allelic richness; *H*_e_, expected heterozygosity; *F*_IS_, inbreeding coefficient).
